# Impact of metformin on hypothalamic–pituitary–thyroid axis activity in women with autoimmune and non-autoimmune subclinical hypothyroidism: a pilot study

**DOI:** 10.1007/s43440-023-00556-3

**Published:** 2023-12-05

**Authors:** Robert Krysiak, Karolina Kowalcze, Bogusław Okopień

**Affiliations:** 1 Department of Internal Medicine and Clinical Pharmacology, Medical University of Silesia, Medyków 18, 40-752 Katowice, Poland; 2 Department of Pediatrics in Bytom, School of Health Sciences in Katowice, Medical University of Silesia, Katowice, Poland

**Keywords:** 5′-Adenosine monophosphate-activated protein kinase, Hypothalamic–pituitary–thyroid axis, Metformin, Thyroid function tests, Thyroiditis

## Abstract

**Background:**

Metformin reduces plasma TSH levels if these levels are elevated. No study has investigated whether the hormonal effects of metformin are impacted by thyroid autoimmunity. The current study aimed to compare the effect of metformin on hypothalamic–pituitary–thyroid axis activity between subjects with mild hypothyroidism of different origins.

**Methods:**

The study population consisted of two groups of women with prediabetes and mildly elevated TSH levels, matched by age, insulin sensitivity, TSH, and thyroid hormone levels. Group A included 26 women with autoimmune thyroiditis, while group B enrolled 26 individuals with hypothyroidism of non-autoimmune origin. Both groups were treated with metformin (2.55–3 g daily). Circulating levels of TSH, total and free thyroid hormones, glucose, insulin, prolactin, high-sensitivity C-reactive protein (hsCRP) and 25-hydroxyvitamin D, concentrations of thyroid antibodies, and structure parameters of thyroid homeostasis were assessed at baseline and 6 months later.

**Results:**

All patients completed the study. At baseline, both groups differed in concentrations of thyroid peroxidase antibodies, thyroglobulin antibodies, hsCRP, and 25-hydroxyvitamin D. The drug reduced TSH and Jostel’s index, with no difference between the study groups. The improvement in insulin sensitivity, observed in both groups, was more pronounced in group B than in group A. In women with autoimmune hypothyroidism, the drug increased SPINA-GT and decreased hsCRP levels. The remaining markers did not change throughout the study.

**Conclusions:**

The obtained results suggest that, despite differences in thyroid output, the impact of metformin on TSH levels is similar in hypothyroid women with and without thyroid autoimmunity.

## Introduction

Because of the lack of the brain–blood barrier [1], single or chronic oral administration of metformin results in higher tissue content of this agent in the pituitary than in the hypothalamus, cerebellum, striatum, frontal cortex, hippocampus and olfactory bulbs [2]. In numerous studies, metformin reduced thyroid-stimulating hormone (TSH) levels if baseline levels of this hormone were elevated [3–6]. This action on overactive thyrotropic cells is in line with the inhibitory effect of metformin on the secretory function of other types of pituitary cells: lactotropes [7, 8] and gonadotropes [9, 10]. However, baseline concentrations do not seem to be the only factor determining the strength of TSH-lowering properties of metformin. The decrease in TSH concentrations was statistically significant in women, but not in men, which may be explained by differences in interaction between this drug and sex hormones at the level of the pituitary gland and/or the hypothalamus [11]. Metformin action may also depend on its tissue content. Although no study assessed the relationship between metformin dose and hypothalamic–pituitary–thyroid axis activity, the impact on prolactin and gonadotropin concentrations was found to be dose-dependent. This effect was significant after treatment with high doses (2.55–3 g daily) of metformin but not in individuals receiving this agent in low doses (1.7 g daily) [8, 10].

Little is known about metformin action in autoimmune (Hashimoto) thyroiditis. A meta-analysis of seven studies conducted by Jia et al. [12] reported that this drug decreased thyroid peroxidase antibody (TPOAb) and thyroglobulin antibody (TgAb) concentrations in individuals (mainly women) with this disorder. Moreover, metformin potentiated a thyroid antibody-lowering effect of levothyroxine in insulin-resistant women [13]. Lastly, metformin/levothyroxine combination therapy was superior to levothyroxine administered together with myo-inositol in reducing thyroid autoimmunity in premenopausal women [14]. These observations are clinically relevant because Hashimoto thyroiditis is the most frequent cause of hypothyroidism in Europe and the United States, and women are 5–10 times more likely to be affected by this disease than men [15, 16].

In an open‐label randomized controlled trial, Palui et al. [17] observed a significantly higher rate of normalization of TSH in thyroid antibody-negative patients than in thyroid antibody-positive patients receiving metformin. This difference was explained by the progressive autoimmune destruction of follicular cells in individuals with autoimmune thyroiditis, and by the impact on the body mass index (BMI) in subjects with non-autoimmune subclinical hypothyroidism. Unfortunately, the study by Palui et al. [17] included individuals of both sexes, as well as both women of reproductive age and after menopause. Moreover, subjects with autoimmune and non-autoimmune hypothyroidism differed in baseline characteristics, and the daily dose of metformin (1.5 g) was lower in comparison with doses exerting a maximum effect on the secretory function of anterior pituitary cells (2.55–3 g daily) [8, 10]. Thus, the current study aimed to investigate whether the impact of metformin, administered at the daily dose of 2.55–3 g, on hypothalamic–pituitary–thyroid axis activity and the calculated parameters of thyroid homeostasis differ between young women with mild subclinical hypothyroidism induced by Hashimoto thyroiditis and unrelated to thyroid autoimmunity.

## Materials and methods

The study protocol was reviewed and approved in advance by the Bioethical Committee of the Medical University of Silesia (Approval No. KNW/0022/KB/161/16; approval date: July 5, 2016), and the study was conducted in accordance with the 1964 Declaration of Helsinki and its subsequent revisions. All patients provided written informed consent after the purpose and procedures of the study had been explained.

### Patients

The participants of this prospective study were recruited among young (18–50 years old) women with untreated prediabetes (fasting plasma glucose concentration at least 100 mg/dL but less than 126 mg/dL, and/or plasma glucose concentration 2 h after a 75-g oral glucose load at least 140 mg/dL but less than 200 mg/dL) resistant to non-pharmacological interventions. They were considered eligible for enrollment if they had TSH levels in the range of 4.5 to 7.5 mU/L, coexisting with plasma total and free thyroid hormones in the reference range (total thyroxine between 55 and 150 nmol/L, total triiodothyronine between 0.9 and 2.8 nmol/L free thyroxine levels between 10.3 and 21.2 pmol/L, and free triiodothyronine between 2.2 and 6.4 pmol/L). No patient considered for enrollment received thyroid hormone substitution. To exclude transient hypothyroidism, TSH and thyroid hormones were re-evaluated 4 weeks later, and patients were included only if both measurements gave similar results. To calculate a baseline value, the results were averaged. The participants (*n* = 52) were assigned to one of two treatment groups. Group A included 26 women with autoimmune thyroiditis, defined as TPOAb concentrations exceeding 100 U/mL and sonographic features of autoimmune thyroiditis (diffuse hypoechoic echogenicity, parenchymal heterogeneity, echogenic septations, hypoechoic micronodularity, and hypervascularity). In turn, group B enrolled 26 subjects with non-autoimmune subclinical hypothyroidism, resulting from partial thyroidectomy (*n* = 15; 58%), non-radical radioiodine therapy (*n* = 2; 8%), thyroid hypoplasia/hemiagenesis (*n* = 5; 19%) and hereditary defects of thyroid hormone synthesis (*n* = 4; 15%). In group B, TPOAb concentrations were within the reference range, and there were no sonographic features of autoimmune thyroid disease. Both groups of women were selected among a greater number of potential participants meeting all inclusion criteria (50 and 52, respectively) to obtain two study groups matched by age, the homeostasis model assessment 1 of insulin resistance index (HOMA1-IR), TSH levels and thyroid hormone levels. To restrict the influence of seasonal confounds and fluctuations in concentrations of 25-hydroxyvitamin D and the remaining variables, similar proportions of patients were recruited in each season: spring (*n* = 14), summer (*n* = 12), fall (*n* = 14), and winter (*n* = 12). The number of patients in each group outnumbered the required sample size. A calculation performed before conducting the study showed that a sample size of 48 participants (24 in each group) was adequate to detect a 20% difference in the primary endpoint (plasma TSH levels).

The exclusion criteria included transient hypothyroidism, diabetes mellitus, other endocrine or autoimmune disorders, cardiovascular disease (except for grade 1 hypertension), impaired renal or hepatic function, malabsorption syndrome, any other serious disorders, pregnancy or lactation, poor patient compliance, and any treatment (including levothyroxine and/or liothyronine).

### Study design

Throughout the study, all participants were treated with metformin. The dose of this agent was slowly increased from a starting dose of 0.85 g once daily in week 1, to 0.85–1.0 g twice daily in weeks 2 and 3, and to a maximum dose of 0.85–1.0 three times a day (2.55–3 g daily), introduced from week 4 onwards. No changes in the treatment regimen were allowed. During the entire study, all participants complied with the goals of lifestyle modification [10]. The patients were seen once a month to ensure adherence to metformin treatment and to boost compliance with the study protocol. Compliance was assessed by interview, tablet count, and analysis of individual dietary questionnaires.

### Laboratory assays

Peripheral venous blood samples were taken from each patient at the beginning of the study and 6 months later. They were collected from the antecubital vein at 8 a.m. after an overnight 12-h fasting in a quiet, temperature-controlled room (23–24 °C). Plasma glucose concentration was assessed using the hexokinase method (Roche Diagnostics, Basel, Switzerland). Plasma levels of TSH, total thyroxine, total triiodothyronine, free thyroxine, free triiodothyronine, insulin, prolactin, and 25-hydroxyvitamin D, as well as concentrations of TPOAb and thyroglobulin antibodies (TgAb) were assayed by direct chemiluminescence using acridinium ester technology (ADVIA Centaur XP Immunoassay System, Siemens Healthcare Diagnostics, Munich, Germany). Plasma hsCRP levels were measured by immunoassay with chemiluminescent detection (Immulite 2000XPi, Siemens Healthcare, Warsaw, Poland). Product codes were as follows: 6491080 (TSH), 9236439 (total thyroxine), 4779663 (total triiodothyronine), 6490106 (free thyroxine), 3154228 (free triiodothyronine), 5481536 (TPOAb), 10492398, (TgAb), 04404483 (glucose), 2230141 (insulin), 9505871 (prolactin), K063057 (hsCRP), and 10631201 (25-hydroxyvitamin D). The homeostasis model assessment 1 of insulin resistance (HOMA1-IR) was calculated as fasting plasma glucose (mg/dL) × fasting plasma insulin (mU/L)/405. Jostel’s TSH index, the structure parameter inference approach (SPINA)-GT index, and the SPINA-GD index were calculated based on TSH and free thyroid hormone levels using SPINA-Thyr 4.0.1 for Windows software in accordance with the formulas described previously [18–20].

### Statistical analysis

Before entering the statistical analysis, all outcome variables were log-transformed to achieve normal distribution and to enable allometric comparisons. The means of variables and the percent changes from baseline in both groups were compared using an unpaired Student's *t* test. The differences between the means of variables within the same treatment group were analyzed with Student’s paired *t* test. Categorical data were analyzed using the *χ*^2^ test. The relationships between the outcome measures were explored by computing bivariate correlations using Pearson's *r* tests. Statistical analyses were performed with the level of significance established at *p* < 0.05.

## Results

Metformin was well tolerated, and no patient was withdrawn from the study because of its adverse effects. All patients complied with treatment recommendations and adhered to recommendations on diet and physical activity. The mean daily dose of this drug did not differ between the groups (2.80 ± 0.20 mg vs. 2.83 ± 0.19 mg; *p* = 0.5818).

At baseline, there were no differences between both groups in age, smoking habits, body mass index (BMI), blood pressure (both systolic and diastolic), TSH, thyroid hormones (free and total), Jostel’s index, SPINA-GT, SPINA-GD, glucose, HOMA1-IR, and prolactin. TPOAb and TgAb concentrations and hsCRP levels were higher in group A than in group B, while the opposite relationship was found for 25-hydroxyvitamin D levels (Tables 1 and 2). Ten out of 26 patients in each group (38%) complained of hypothyroid symptoms (general fatigue, constipation, dry skin, edema, weight gain, and/or hoarseness).

**Table 1:** Baseline characteristics of patients

Variable	Group A	Group B	*t* value/*χ*^2^-value	*p* value
Number (*n*)	26	26	–	–
Age (years)	37 ± 7	36 ± 7	*t*_50_ = 0.515	0.6088
Smokers: total/current/ex– (%)	35/27/8	38/30/8	*χ*^2^ = 1.95 *df* = 2	0.3458
Number of cigarettes a day (*n*)	10 ± 6	12 ± 6	*t*_50_ = 1.202	0.2351
Duration of smoking (months)	142 ± 50	144 ± 48	*t*_50_ = 0.147	0.8836
BMI (kg/m^2^)	24.8 ± 5.0	24.5 ± 5.2	*t*_50_ = 0.212	0.8329
Systolic blood pressure (mmHg)	134 + 14	132 ± 17	*t*_50_ = 0.463	0.6453
Diastolic blood pressure (mmHg)	86 ± 7	85 ± 5	*t*_50_ = 0.593	0.5560

Group A: women with autoimmune thyroiditis

Group B: women without autoimmune thyroid disease

Unless otherwise stated, the data are presented as the mean ± standard deviation. The means of variables in both groups were compared using an unpaired Student's *t* test. Categorical variables were compared using the *χ*^2^ test. Statistical significance was defined as a *p* value below 0.05

BMI, body mass index

**Table 2:** The effect of metformin on the investigated variables in the study population

Variable	Group A	Group B	Group A versus Group B	
			*t* value	*p* value
TSH (mU/L)				
Baseline	6.2 ± 0.8	6.0 ± 0.8	*t*_50_ = 0.901	0.3717
Follow-up	4.7 ± 1.0	4.8 ± 1.2	*t*_50_ = 0.326	0.7455
*t* value (follow-up vs. baseline)	*t*_25_ = 5.972	*t*_25_ = 4.243	–	–
*p* value (follow-up vs. baseline)	< 0.0001	0.0001	–	*–*
Total thyroxine (nmol/L)				
Baseline	85 ± 18	88 ± 20	*t*_50_ = 0.569	0.5722
Follow-up	91 ± 22	95 ± 24	*t*_50_ = 0.626	0.5506
*t* value (follow-up vs. baseline)	*t*_25_ = 1.076	*t*_25_ = 1.143	–	–
*p* value (follow-up vs. baseline)	0.2870	0.2587	–	–
Total triiodothyronine (nmol/L)				
Baseline	1.6 ± 0.4	1.7 ± 0.5	*t*_50_ = 0.796	0.4296
Follow-up	1.8 ± 0.5	1.9 ± 0.6	*t*_50_ = 0.653	0.5188
*t* value (follow-up vs. baseline)	*t*_25_ = 1.692	*t*_25_ = 1.406	–	–
*p* value (follow-up vs. baseline)	0.1175	0.1976	–	–
Free thyroxine (pmol/L)				
Baseline	14.8 ± 2.0	15.1 ± 2.3	*t*_50_ = 0.502	0.6180
Follow-up	16.1 ± 2.8	15.2 ± 3.0	*t*_50_ = 1.118	0.2688
*t* value (follow-up vs. baseline)	*t*_25_ = 1.980	*t*_25_ = 0.143	–	–
*p* value (follow-up vs. baseline)	0.0597	0.8932	–	–
Free triiodothyronine (pmol/L)				
Baseline	3.2 ± 0.7	3.2 ± 0.6	*t*_50_ = 0.000	1.0000
Follow-up	3.6 ± 0.9	3.5 ± 0.8	*t*_50_ = 0.423	0.6738
*t* value (follow-up vs. baseline)	*t*_25_ = 1.689	*t*_25_ = 1.556	–	–
*p* value (follow-up vs. baseline)	0.1137	0.1324	–	–
TPOAb (IU/mL)				
Baseline	925 ± 288	18 ± 10	*t*_50_ = 16.049	< 0.0001
Follow-up	780 ± 235	16 ± 9	*t*_50_ = 16.565	< 0.0001
*t* value (follow-up vs. baseline)	*t*_25_ = 1.998	*t*_25_ = 0.758	–	–
*p* value (follow-up vs. baseline)	0.0521	0.4520	–	–
TgAb (IU/mL)				
Baseline	835 ± 342	16 ± 8	*t*_50_ = 12.207	< 0.0001
Follow-up	675 ± 240	14 ± 8	*t*_50_ = 14.036	< 0.0001
*t* value (follow-up vs. baseline)	*t*_25_ = 1.972	*t*_25_ = 0.901	–	–
*p* value (follow-up vs. baseline)	0.0565	0.3718	–	–
Jostel’s index				
Baseline	3.8 ± 0.2	3.8 ± 0.2	*t*_50_ = 0.000	1.0000
Follow-up	3.6 ± 0.2	3.6 ± 0.2	*t*_50_ = 0.000	1.0000
*t* value (follow-up vs. baseline)	*t*_25_ = 3.606	*t*_25_ = 2.828	–	–
*p* value (follow-up vs. baseline)	0.0007	0.0067	–	–
SPINA-GT (pmol/s)				
Baseline	1.62 ± 0.28	1.69 ± 0.32	*t*_50_ = 0.839	0.4052
Follow-up	1.94 ± 0.21	1.81 ± 0.24	*t*_50_ = 2.087	0.0428
*t* value (follow-up vs. baseline)	*t*_25_ = 4.662	*t*_25_ = 1.530	–	–
*p* value (follow-up vs. baseline)	< 0.0001	0.1324	–	–
SPINA-GD (nmol/s)				
Baseline	19.98 ± 2.52	19.64 ± 3.26	*t*_50_ = 0.421	0.6757
Follow-up	20.68 ± 3.14	21.29 ± 3.96	*t*_50_ = 0.615	0.5410
*t* value (follow-up vs. baseline)	*t*_25_ = 0.887	*t*_25_ = 1.640	–	–
*p* value (follow-up vs. baseline)	0.3796	0.1102	–	–
Glucose (mgl/L)				
Baseline	110 ± 9	111 ± 10	*t*_50_ = 0.379	0.7063
Follow-up	103 ± 11	102 ± 10	*t*_50_ = 0.341	0.7324
*t* value (follow-up vs. baseline)	*t*_25_ = 2.585	*t*_25_ = 3.245	–	–
*p* value (follow-up vs. baseline)	0.0153	0.0050	–	–
HOMA1-IR				
Baseline	3.8 ± 1.2	3.9 ± 1.4	*t*_50_ = 0.277	0.7834
Follow-up	3.0 ± 1.1	2.0 ± 0.8	*t*_50_ = 3.749	0.0005
*t* value (follow-up vs. baseline)	*t*_25_ = 2.578	*t*_25_ = 6.008	–	–
*p* value (follow-up vs. baseline)	0.0155	< 0.0001	–	–
Prolactin (ng/mL)				
Baseline	22.4 ± 6.8	21.7 ± 7.0	*t*_50_ = 0.366	0.7161
Follow-up	19.6 ± 6.0	18.9 ± 7.4	*t*_50_ = 0.375	0.7095
*t* value (follow-up vs. baseline)	*t*_25_ = 1.574	*t*_25_ = 1.402	–	–
*p* value (follow-up vs. baseline)	0.1217	0.1672	–	–
hsCRP (mg/L)				
Baseline	2.8 ± 1.2	1.6 ± 0.8	*t*_50_ = 4.243	0.0001
Follow-up	2.1 ± 0.8	1.5 ± 0.7	*t*_50_ = 2.878	0.0059
*t* value (follow-up vs. baseline)	*t*_25_ = 2.504	*t*_25_ = 0.480	–	–
*p* value (follow-up vs. baseline)	0.0168	0.6440	–	–
25-Hydroxyvitamin D (nmol/L)				
Baseline	55.1 ± 14.0	69.2 ± 18.0	*t*_50_ = 3.152	0.0027
Follow-up	60.2 ± 15.8	73.1 ± 19.5	*t*_50_ = 2.621	0.0116
*t* value (follow-up vs. baseline)	*t*_25_ = 1.232	*t*_25_ = 0.789	–	–
*p* value (follow-up vs. baseline)	0.2238	0.4572	–	–

Group A: women with autoimmune thyroiditis

Group B: women without autoimmune thyroid disease

The data are presented as the mean ± standard deviation. The means of variables in both groups were compared using an unpaired Student's *t* test. The differences within the same treatment group were analyzed with Student’s paired *t* test. Statistical significance was defined as a *p* value below 0.05

Reference values: TSH: 0.4–4.5 mU/L; total thyroxine: 55–150 nmol/L; total triiodothyronine: 0.9–2.8 nmol/L; free thyroxine: 2.2 and 6.4 pmol/L; free triiodothyronine 2.2–6.4 pmol/L; TPOAb: < 35 U/mL; TgAb: < 35 U/mL; Jostel’s index: 1.3–4.1; SPINA-GT: 1.4–8.7 pmol/s; SPINA-GD: 20–60 nmol/s; glucose: 70–99 mg/dL, HOMA1-IR: < 2.0; prolactin: 5.0–25.0 ng/mL;; hsCRP: < 1.0 mg/L, 25-hydroxyvitamin D: 75–150 nmol/L

HOMA1-IR, the homeostasis model assessment 1 of insulin resistance index; hsCRP, high-sensitivity C-reactive protein; SPINA, structure parameter inference approach; TgAb, thyroglobulin antibodies; TPOAb, thyroid peroxidase antibodies; TSH, thyroid-stimulating hormone

In both groups, the drug reduced TSH, Jostel’s index, glucose, and HOMA1-IR. Only in group A, did the drug increase SPINA-GT and decreased hsCRP levels. Total thyroxine, total triiodothyronine, free thyroxine, free triiodothyronine, TPOAb, TgAb, SPINA-GD, prolactin, and 25-hydroxyvitamin D remained at a similar level throughout the study period. There were differences between the study groups in follow-up values of antibody concentrations, SPINA-GT, HOMA1-IR, hsCRP, and 25-hydroxyvitamin D (Table 2). Both groups differed in the percent changes from baseline in free thyroxine, SPINA-GT, HOMA1-IR, and hsCRP (Table 3). In neither treatment group did metformin affect BMI and blood pressure (data not shown).

**Table 3:** Percent changes from baseline in the investigated variables in the study population

Variable	Group A	Group B	*t* value	*p* value
Δ TSH	− 24 ± 10	− 20 ± 8	*t*_50_ = 1.593	0.1175
Δ Total thyroxine	7 ± 5	8 ± 6	*t*_50_ = 0.653	0.5168
Δ Total triiodothyronine	13 ± 8	12 ± 6	*t*_50_ = 0.510	0.6124
Δ Free thyroxine	9 ± 12	1 ± 10	*t*_50_ = 2.611	0.0119
Δ Free triiodothyronine	13 ± 10	9 ± 8	*t*_50_ = 1.593	0.1175
Δ TPOAb	− 16 ± 11	− 11 ± 12	*t*_50_ = 1.566	0.1236
Δ TgAb	− 19 ± 14	− 13 ± 18	*t*_50_ = 1.342	0.1858
Δ Jostel’s index	− 5 ± 5	− 5 ± 6	*t*_50_ = 0.000	1.0000
Δ SPINA-GT	20 ± 10	7 ± 8	*t*_50_ = 5.176	< 0.0001
Δ SPINA-GD	4 ± 10	8 ± 13	*t*_50_ = 1.244	0.2195
Δ Glucose	− 6 ± 5	− 8 ± 5	*t*_50_ = 1.442	0.1555
Δ HOMA1-IR	− 21 ± 12	− 49 ± 20	*t*_50_ = 6.121	< 0.0001
Δ Prolactin	− 13 ± 8	− 13 ± 10	*t*_50_ = 0.000	1.0000
Δ hsCRP	− 25 ± 12	− 6 ± 7	*t*_50_ = 6.974	< 0.0001
Δ 25-hydroxyvitamin D	9 ± 10	6 ± 5	*t*_50_ = 1.368	0.1774

Group A: women with autoimmune thyroiditis

Group B: women without autoimmune thyroid disease

The data are presented as the mean ± standard deviation. Percent changes from baseline in both groups were compared using an unpaired Student's *t* test. Statistical significance was defined as a *p* value below 0.05

HOMA1-IR, the homeostasis model assessment 1 of insulin resistance index; hsCRP, high-sensitivity C-reactive protein; SPINA, structure parameter inference approach; TgAb, thyroglobulin antibodies; TPOAb, thyroid peroxidase antibodies; TSH, thyroid-stimulating hormone

**Fig. 1:**
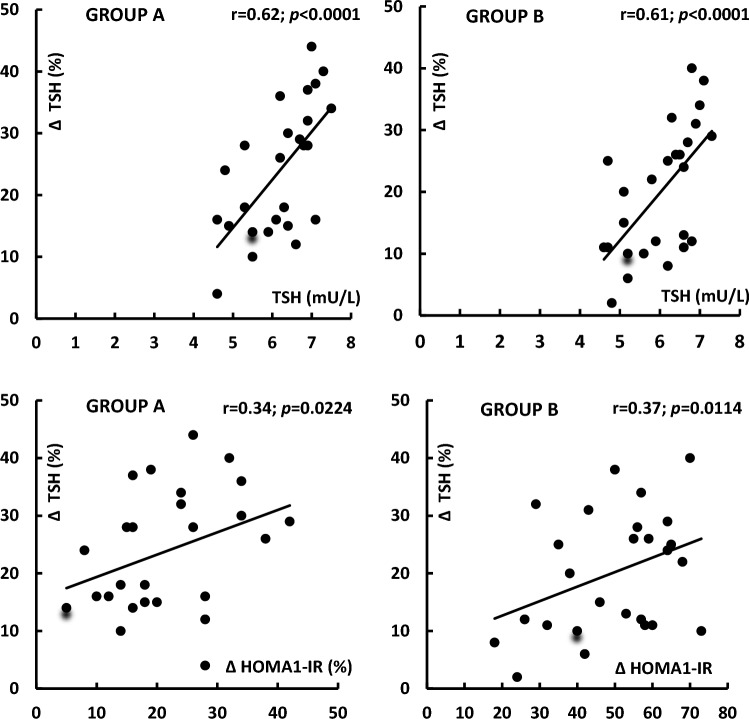
Correlations between the impact of metformin on TSH levels and baseline TSH levels and metformin-induced changes in HOMA1-IR. Correlations were estimated using Pearson’s r tests*.* Statistical significance was defined as a *p* value below 0.05. *Abbreviations*: HOMA1-IR, the homeostasis model assessment 1 of insulin resistance index; TSH, thyroid-stimulating hormone

**Fig. 2:**
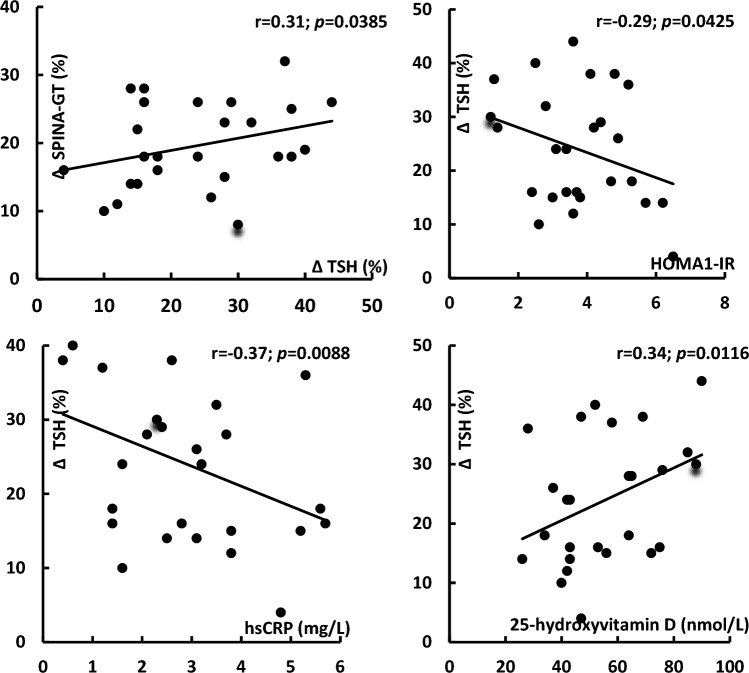
Correlations between the impact of metformin on TSH levels and on SPINA-GT, and between the impact of metformin on TSH levels and baseline values of HOMA1-IR, hsCRP and 25-hydroxyvitamin D in women with mild subclinical hypothyroidism induced by autoimmune thyroiditis. Correlations were estimated using Pearson’s r tests*.* Statistical significance was defined as a *p* value below 0.05. *Abbreviations*: HOMA1-IR, the homeostasis model assessment 1 of insulin resistance index; hsCRP, high-sensitivity C-reactive protein; SPINA, structure parameter inference approach; TSH, thyroid-stimulating hormone

**Fig. 3:**
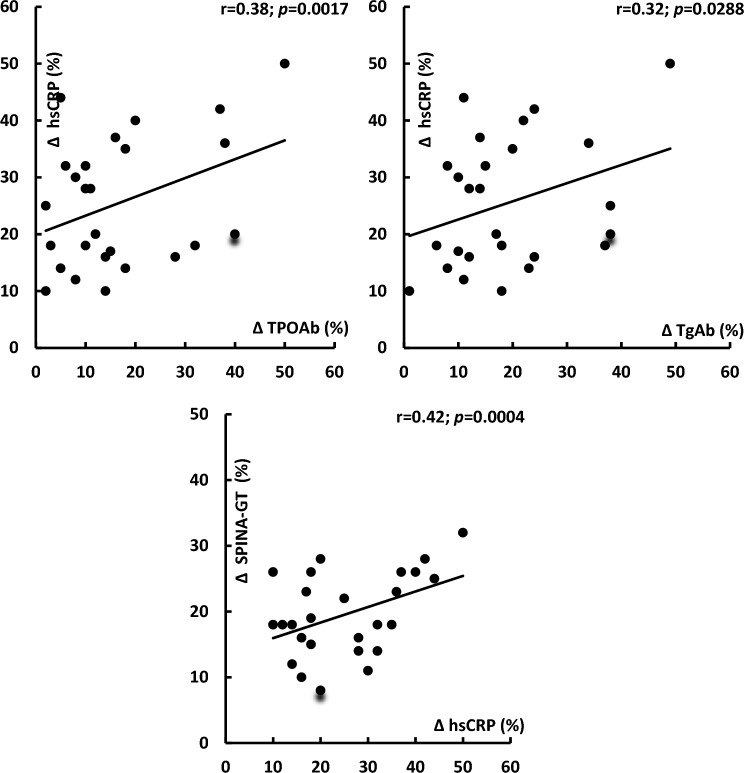
Correlations between the impact of metformin on hsCRP levels and metformin-induced changes in thyroid antibodies and SPINA-GT in women with mild subclinical hypothyroidism induced by autoimmune thyroiditis. Correlations were estimated using Pearson’s r tests*.* Statistical significance was defined as a *p* value below 0.05. *Abbreviations*: hsCRP, high-sensitivity C-reactive protein; SPINA, structure parameter inference approach; TgAb, thyroglobulin antibodies; TPOAb, thyroid peroxidase antibodies

At baseline, hsCRP levels in group A positively correlated with TPOAb (*r* = 0.40, *p* = 0.0004) and TgAb (*r* = 0.35, *p* = 0.0128). In both study groups, treatment-induced changes in TSH levels correlated with their baseline values and with the impact of treatment on HOMA1-IR (Fig. 1). In group A, the decrease in TSH positively correlated with the increase in SPINA-GT and baseline 25-hydroxyvitamin D levels, as well as inversely correlated with baseline values of HOMA1-IR and hsCRP (Fig. 2). In this group of patients, there was also positive correlation between treatment-induced changes in hsCRP and the impact of metformin on TPOAb, TgAb and SPINA-GT (Fig. 3). All other correlations did not reach the level of statistical significance.

## Discussion

Although subclinical hypothyroidism is characterized by TSH above the upper limit of normal coexisting with normal free thyroxine [21, 22], our study included only patients with TSH levels not exceeding 7.5 mU/L. This cut-off value was selected to not enroll subjects requiring levothyroxine therapy. Although indications for thyroid hormone substitution are TSH concentrations above 10 mIU/L [21, 23], levothyroxine treatment may or should be considered in patients with milder forms of hypothyroidism (repeated measurements of TSH between the upper limit of normal and 10 mIU/L), if they have symptoms suggestive of hypothyroidism, are TPOAb-positive or plan to conceive [22, 24]. Because the percentage of symptomatic patients with subclinical hypothyroidism and the severity of these symptoms correlate with TSH levels [23], the probability of thyroid hormone substitution is lower in patients with TSH concentrations between 4.5 and 7.5 mIU/L than in individuals with TSH above 7.5 mIU/L.

In the current study, metformin reduced TSH levels to a similar degree in levothyroxine-naïve patients with mild subclinical hypothyroidism of both autoimmune and non-autoimmune origin. Moreover, in both study groups, the drug decreased Jostel’s index, a calculated structure parameter of thyroid homeostasis estimating the thyrotropic function of the anterior pituitary lobe [18]. These changes, no between-group differences in follow-up values of TSH and Jostel’s index, as well as strong correlations between changes in TSH levels and baseline levels of this hormone show that the impact of metformin on TSH is determined mainly by the degree of overactivity of pituitary thyrotropes, and that this effect does not seem to be impacted by thyroid autoimmunity. Thyrotropes are one of two populations of anterior pituitary cells (besides gonadotropes) with the highest expression of 5′-adenosine monophosphate-activated protein kinase (AMPK) [25], which is a master regulator of cellular energy homeostasis [26] and mediates many biological effects of metformin, including the impact on glucose homeostasis and the decrease in gonadotropin secretion [25, 27]. Interestingly, metformin-induced reduction in TSH levels correlated with the improvement in insulin sensitivity. Thus, it seems that AMPK may, at least partially, mediate metformin action on TSH secretion.

Another interesting finding of the current study is differences in the impact of metformin on thyroid output. In women with autoimmune thyroiditis, but not in women with mild hypothyroidism secondary to partial thyroidectomy, non-radical radioiodine therapy, thyroid hypoplasia/hemiagenesis or dyshormonogenesis, the drug increased SPINA-GT, a parameter estimating the maximum secretion rate of the thyroid gland under stimulated conditions [19, 20]. This observation indicates that in women with Hashimoto thyroiditis, but not in other groups of hypothyroid women, metformin may activate hypothalamic–pituitary–thyroid axis activity directly at the level of thyrocytes. However, considering unaltered fasting thyroid hormone levels, it seems that this effect is weak. This finding probably reflects the anti-inflammatory properties of the studied drug because metformin decreased levels of hsCRP, which is considered a marker of systemic inflammation [28]. Relatively mild immunosuppressive properties of metformin and the small sample size probably explain why the impact on TPOAb and TgAb did not reach the level of statistical significance, though statistically significant changes in antibody concentrations were reported in a meta-analysis of seven interventional studies (most of which were carried out by our research team) [12]. The role of anti-inflammatory action in the impact of metformin on thyrocytes is also supported by the presence of correlations between the increase in SPINA-GT and the degree of reduction in hsCRP, as well as between hsCRP and thyroid antibodies. In our previous study [29], women with euthyroid autoimmune thyroiditis were characterized by elevated hsCRP levels, correlating with monocyte and lymphocyte production of proinflammatory cytokines. Interestingly, metformin was found to decrease levels of proinflammatory cytokines, including those assessed by our research group (interleukin-1ß, tumor necrosis factor-α, and interleukin-6) [30, 31]. No alteration in both total and free thyroid hormones excludes the association between changes in thyroid output and the impact on thyroid hormone protein binding. In turn, no effect on SPINA-GD, estimating the sum activity of peripheral deiodinases [19, 20], is an argument against the association with differences in conversion to triiodothyronine. Low values of this parameter in both groups over the entire study period probably reflect low selenium status [32]. Deiodinases, enzymes converting thyroxine to triiodothyronine, are selenoproteins, and decreased selenium availability reduces their activity [33]. Thus, metformin administered to patients with mild autoimmune subclinical hypothyroidism may increase thyroid output by a reduction in thyroid lymphocytic infiltration and/or partial restoration of secretory function of preserved thyrocytes, which is inhibited by proinflammatory cytokines and other inflammatory mediators [34].

An increase in SPINA-GT, correlating with the changes in TSH levels, only in women with Hashimoto thyroiditis was in contradiction with a similar decrease in TSH and Jostel’s index in both study populations. No between-group difference in the impact on thyrotrope secretory function, despite the improvement in thyroid output only in one study group suggests that the positive feedback between changes in thyroid's secretory capacity and TSH secretion might have been counterbalanced by the negative feedback between TSH and other factors. In women with autoimmune thyroid disease, but not in women with non-autoimmune hypothyroidism, metformin-induced changes in TSH inversely correlated with hsCRP levels, and positively correlated with 25-hydroxyvitamin D concentrations, which is in line with this explanation. Thus, systemic inflammation and low vitamin D status, characterizing women with Hashimoto thyroiditis, may impair the response of thyrotropic cells to increased thyroid output. This interaction is likely to take place at the level of the pituitary AMPK because inflammation and vitamin D insufficiency were found to inhibit the AMPK pathway [35, 36], whereas the opposite effect was exerted by thyroid hormones [37].

We have also observed between-group differences in the impact of metformin on HOMA1-IR. Although present in women with hypothyroidism of different origins, the improvement in insulin sensitivity was less pronounced if subclinical hypothyroidism was induced by thyroid autoimmunity. This finding may be explained by interactions between inflammation and metformin at the level of the glucose transporter type 4 in the skeletal muscles, adipose tissue, and other peripheral tissues. The glucose transporter type 4 is the most important glucose transporter and plays a fundamental role in cellular glucose uptake in response to insulin [38]. In line with our explanation, its translocation and membrane expression were blocked by proinflammatory cytokines associated with thyroid autoimmunity: interleukin-1ß, tumor necrosis factor-α, and interferon-γ [39–41].

We can only speculate about the clinical relevance of the obtained results. Although some researchers regard mildly elevated TSH levels as a compensatory state enabling to maintain of normal thyroid hormone production, the majority of investigators consider persistent isolated hyperthyrotropinemia as a marker of mild thyroid hormone deficiency [21, 23]. The decrease in TSH and Jostel’s index, which was not accompanied by changes in thyroid hormone levels, indirectly argues against the first explanation. Our findings indicate that metformin treatment, though may make subclinical hypothyroidism more difficult to detect, does not seem to worsen thyroid function. Thus, this drug can be safely used in the treatment of glucose metabolism abnormalities in patients who have concomitant hypothyroidism. What is more, despite a decrease in TSH, women with autoimmune subclinical hypothyroidism may have slightly improved thyroid secretory function. It needs to be investigated whether this effect is stronger and results in an increase in circulating levels of thyroid hormones if metformin is administered together with other agents found to increase the secretory capacity of the thyroid gland in young women with autoimmune thyroiditis (vitamin D, selenomethionine and myo-inositol) [42]. Because both persistently raised TSH concentrations [43] and insulin resistance [44, 45], often diagnosed in the same person [46, 47], were found to predispose to an increase in thyroid size and the development of non-toxic nodular goiter, metformin may have a protective effect against thyroid enlargement and the development of nodular thyroid disease in hypothyroid patients with impaired glucose homeostasis. Because we did not assess the impact of metformin on clinical symptoms, quality of life, and peripheral markers of thyroid hormone action, the question of whether metformin treatment ameliorates symptoms of hypothyroidism remains unanswered, and therefore it is too early to state whether this drug may be used in patients with this disorder who cannot receive levothyroxine.

To make the population more homogeneous, the study purposely included only women of reproductive age. Several reasons justified the choice of this population. The majority of studies reporting the decrease in TSH in response to metformin treatment included only or predominantly women, often of reproductive age [3–6]. The impact of metformin on thyrotrope function was found to be stronger in females than in males [11]. A positive association with estrogen production and a negative association with androgen production [11] suggest that the impact of metformin on thyrotrope secretory function may be more pronounced in young women than in men and postmenopausal women. Lastly, this group of patients is generally healthier than older subjects, usually without comorbidities, rarely treated with other drugs, and, as our previous studies [13, 14] showed, highly compliant with medication use. Although the obtained results can only apply to young women, they encourage comparing metformin action on hypothalamic–pituitary–thyroid axis activity between autoimmune and non-autoimmune hypothyroidism also in other demographic groups.

The strength of the obtained results includes two matched populations of women with mildly elevated TSH. Thus, our results cannot be explained by the impact of age, body mass, insulin sensitivity, and baseline activity of the hypothalamus–pituitary–thyroid axis, which were similar in both groups of women. Owing to strict inclusion and exclusion criteria they cannot be also associated with coexisting disorders, particularly other autoimmune disorders, often present in individuals with Hashimoto thyroiditis [15, 16], or with concurrent therapies that may affect thyrotrope secretory function by themselves or by pharmacokinetic or pharmacodynamic interactions with metformin.

Some study shortcomings bear mentioning. A limited number of patients and a short observation period make drawing any strong conclusions difficult. Because the study was non-randomized and did not include a group of placebo-treated women, the obtained results might have been influenced by latent confounders and selection bias. We assume (extrapolating data from previous studies [32, 48]) that the participants were characterized by low selenium status and adequate iodine intake. Thus, it is uncertain whether the impact of metformin on hypothalamic–pituitary–thyroid axis activity is the same in populations with sufficient selenium and/or inadequate iodine intake. Lastly, the obtained results do not allow us to conclude about the association between metformin action and thyroid autoimmunity in patients on levothyroxine replacement therapy.

In conclusion, metformin reduced TSH concentrations in young prediabetic women with subclinical hypothyroidism, with no difference between hypothyroidism of autoimmune and non-autoimmune origin, likely by inhibiting the secretory function of thyrotropic cells. Although a weaker insulin-sensitizing effect than in women with non-autoimmune subclinical disease suggests that this agent may be a less effective diabetes prevention strategy in case of concurrent thyroid autoimmunity, young women with autoimmune hypothyroidism may also benefit from treatment with metformin. A decrease in systemic inflammation related to autoimmune thyroid disease and an increase in thyroid output suggest the anti-inflammatory effects of metformin in this group of patients, and its possible role in delaying the development of full-blown hypothyroidism in young women with Hashimoto thyroiditis. Novelty of our findings and the limitations of the study design cause that they need to be confirmed in larger scale prospective studies. The obtained results should be also an incentive to compare the impact of metformin on hypothalamic–pituitary–thyroid axis activity in other groups of patients (men, postmenopausal women, and levothyroxine-treated patients).

## Data Availability

The datasets generated during and/or analyzed during the current study are available from the corresponding author upon reasonable request.

## Abbreviations

AMPK: 5′-Adenosine monophosphate-activated protein kinase
BMI: Body mass index
HOMA1-IR: The homeostasis model assessment 1 of insulin resistance index
hsCRP: High-sensitivity C-reactive protein
SPINA: Structure parameter inference approach
TgAb: Thyroglobulin antibodies
TPOAb: Thyroid peroxidase antibodies
TSH: Thyroid-stimulating hormone

## Declarations

### Institutional approval

The study was approved by the institutional review board.
